# Benzoquinones from scent glands of phalangiid harvestmen (Arachnida, Opiliones, Eupnoi): a lesson from *Rilaena triangularis*

**DOI:** 10.1007/s00049-014-0177-y

**Published:** 2014-10-24

**Authors:** Günther Raspotnig, Miriam Schaider, Petra Föttinger, Verena Leutgeb, Christian Komposch

**Affiliations:** 1 Institute of Zoology, University of Graz, Universitätsplatz 2, 8010 Graz, Austria; 2 Research Unit of Osteology and Analytical Mass Spectrometry, Medical University, University Children’s Hospital, Auenbruggerplatz 30, 8036 Graz, Austria; 3 Department of Limnology and Bio-Oceanography, University of Vienna, Althanstraße 14, 1090 Vienna, Austria; 4 Institute of Animal Ecology and Landscape Planning, ÖKOTEAM, Bergmanngasse 22, 8010 Graz, Austria

**Keywords:** Scent glands, Chemical defense, Benzoquinone, Naphthoquinone, Caprylic acid, Palpatores, Eupnoi

## Abstract

In case of disturbance, the phalangiine harvestman *Rilaena triangularis* (Eupnoi, Phalangiidae) emits a directed jet from large prosomal scent (“defensive”) glands. The pungent-smelling secretion was analyzed by gas chromatography–mass spectrometry and found to contain mainly 1,4-benzoquinone along with 1,4-naphthoquinone and caprylic (=octanoic) acid. While various alkylated benzoquinones are characteristic for the scent gland secretions of many grassatorean Laniatores, this is the first incidence of benzoquinone-based chemical defense in palpatorean harvestmen.

## Introduction

The scent glands of harvestmen are well known for the production of odoriferous secretions that serve in chemical defense against predators (e.g., Eisner et al. 2004; Machado et al. 2005), but also in microbial protection (e.g., Fieser and Ardao 1956; Rocha et al. 2013) and chemical communication (e.g., Holmberg 1986; Machado et al. 2002). Moreover, scent gland secretions represent a stunningly diverse source of natural products, including rare naphthoquinones and methyl ketones in Cyphophthalmi (Raspotnig et al. 2005, 2012; Jones et al. 2009), nitrogen-containing compounds in travunioid Insidiatores (Ekpa et al. 1984; Raspotnig et al. 2011), and alkylated phenols in some Grassatores (Shear et al. 2010a, b; Pomini et al. 2010). The best-known chemical class of scent gland-derived products, however, is certainly the benzoquinones which have been documented for many Grassatores of superfamily Gonyleptoidea (Hara et al. 2005; Caetano and Machado 2013; Raspotnig et al. 2014b). In detail, benzoquinones were found to prevail the secretions of gonyleptoid families, Cosmetidae, Manaosbiidae, and Gonyleptidae (e.g., Hara et al. 2005; Föttinger et al. 2010), but have not yet been reported from the secretions of any other major harvestman clade. Indeed, an assumed chemical dichotomy between the benzoquinone-rich exudates from gonyleptoid Laniatores and the non-benzoquinone chemistry of the remaining Opiliones represented an early paradigm of harvestman chemosystematics (e.g., Roach et al. 1980; Duffield et al. 1981).

However, large gaps in the knowledge of opilionid scent gland chemistry still exist, and the largest gap certainly refers to the Palpatores—a species-rich group of “daddy-long legs” from which more than 2,000 representatives have so far been described. The Palpatores can be classed into two large groups, often given the rank of suborders, namely the Dyspnoi (about 330 species) and the Eupnoi (about 1,820 species) (Kury 2013). The phylogenetic relationship between Dyspnoi and Eupnoi is still being discussed, either considering them as sister groups, resulting in monophyletic Palpatores, or regarding Dyspnoi as the sister group of Laniatores, leading to the so-called Dyspnolaniatores hypothesis (Machado et al. 2007). Anyway, apart from few studies on their scent gland morphology (Juberthie et al. 1991; Holmberg 1970; Lopez et al. 1980; Clawson 1988; Schaider and Raspotnig 2009), data on palpatorean scent glands, particularly on the chemistry of secretions, are scarce. The secretion chemistry of only 15 species (=less than 1 % of described Palpatores) has so far been investigated. Indeed, scent glands in Palpatores appear to be generally less developed and more heterogenous than in representatives of Cyphophthalmi and Laniatores; they may even represent “cryptic” organs with hidden or covered orifices such as in certain taxa of Dyspnoi (Schaider and Raspotnig 2009). Also, the mode of secretion discharge varies from emission of forceful jets or sprays to not observable or even no discharge at all (e.g., Pabst 1953; Gnaspini and Hara 2007). These aberrant conditions led to many methodological problems with respect to the extraction of glandular contents (Raspotnig et al. 2014a), resulting in decreasing attention on palpatorean scent glands during the last decades.

However, initial chemical data indicate chemically diverse secretions, in any case representing relevant data for a consistent chemosystematic overall picture of the Opiliones. Regarding the Dyspnoi, scent gland chemistry has eventually been studied on a few representatives of Nemastomatidae, demonstrating the presence of naphthoquinones, methoxy-naphthoquinones and anthraquinones in *Paranemastoma quadripunctatum* (Raspotnig et al. 2010) and naphthoquinones along with highly volatile ketones in the species of *Carinostoma* (Raspotnig et al. 2014a). The little that is known on Eupnoi scent gland chemistry is still restricted to investigations from the 1970s and 1980s and refers to an extremely small taxonomic unit of nine North American species of the sclerosomatid genus *Leiobunum*, one species of *Hadrobunus* (both genera in Leiobuninae) as well as one single representative of family Phalangiidae, *Phalangium opilio*. While the secretions of leiobunine Sclerosomatidae contained a variety of acyclic ethyl ketones and biosynthetically related compounds (review in Ekpa et al. 1985), *P. opilio* exclusively produced naphthoquinones (Wiemer et al. 1978).

Here, we report on the scent gland secretion of a second phalangiid, *Rilaena triangularis*, that produces a mixture of benzo- and naphthoquinones. Benzoquinones—so far considered characteristic of scent glands of gonyleptoid Laniatores only—are introduced as a third class of compounds to the secretion chemistry of Eupnoi. We furthermore provide evidence for the multiple independent evolution of benzoquinones in the scent glands of Opiliones.

## Materials and methods

### Collection of specimens

In total, 183 individuals of *Rilaena triangularis* (Herbst, 1799) were collected by hand at different localities in South-Eastern Austria (mainly Carinthia and Styria: Table 1). This species is widespread in Europe, ranging from Western and Central Europe to Southern Scandinavia, Russia and southwards to the northern parts of the Balkan Peninsula. Its vertical distribution reaches from the lowlands to the montane level in the Alps. Habitats are forests, flood-plain forests and meadowsweet fens (Martens 1978; Komposch and Gruber 2004). Juveniles live in the soil layer whereas adults can be found on low vegetation in spring, and later on tree trunks up to treetops. We used adults of both sexes as well as juveniles for our chemical study.

**Table 1:** Collection of *Rilaena triangularis*

Location (in Austria)	Geographical co-ordinates (altitude)	Date	Individuals
Carinthia, near Ferlach, Osce	N 46°31′32′′, E 14°19′10′′ (475 m)	24 February 2008	2 juv
		22 March 2009	3 juv
Carinthia, near Ferlach, Rauth	N 46°31′32′′, E 14°19′32′′ (453 m)	12 May 2012^b^	2 ♂, 8 ♀
	N 46°31′29′′, E 14°19′38′′ (462 m)	9 June 2012^b^	6 ♂, 6 ♀
		24 June 2012	2 ind^a^
Styria, Graz, Berliner Ring	N 47°04′37′′, E 15°29′42′′ (398 m)	6 May 2009	3 ♀
		7 May 2009	3 ♂, 4 ♀
Styria, Graz, Hilmteich	N 47°05′04′′, E 15°27′43′′ (400 m)	6 May 2012	2 ♂, 3 ♀
		10 May 2012	5 ♂, 7 ♀, + 1 ind^a^
		23 May 2012	2 ♂, 4 ♀
		24 May 2012	4 ♂, 4♀
		25 May 2012	8 ♂, 4 ♀, 1 iuv
		30 May 2012	6 ind^a^
		7 June 2012	2 ♂
Styria, Graz, Maria Trost	N 47°06′32′′, E 15°29′39′′ (437 m)	22 November 2011	2 juv
		18 April 2012	2 juv
Styria, Graz, Plabutsch	N 47°05′, E 15°23′ (430 m)	24 April 2008	1♀
		11 June 2008	4 ♂
Styria, Graz, Platte	N 47°06′52′′, E 15°28′22′′ (557 m)	10 May 2011	1 ♂
Styria, Graz, Rosenhain	N 47°05′, E 15°26′ (400 m)	5 November 2009	1 juv
		28 May 2010	1 ♂, 1 ♀
		29 May 2010	2 ♀
		7 June 2010	1 ♂
		15 June 2010	1 ♀
		18 June 2010	1 ♂
		22 June 2010	3 ♂
		6 June 2011	1 ♂
		16 June 2013^b^	3 ind^a^
		22 June 2013^b^	7 ind^a^
Styria, near Graz, Kroisbach	N 47°05′, E 15°28′ (390 m)	22 June 2010	1 ♂
		30 March 2011	1 juv
		5 April 2012	5 juv
		23 April 2012	5 juv
Styria, near Graz, Ragnitz, Ragnitzbach	N 47°04′, E 15°29′ (390 m)	6 May 2009	2 ♀
Styria, near Graz, Rein, Pleschwald	N 47°08′, E 15°16′ (631 m)	5 April 2009	9 juv
Styria, Deutschlandsberger Klause, Hochwald	N 46°48′, E 15°11′ (807 m)	29 May 2009	3 ♂, 2 ♀
		3 May 2012	2 ♂, 1 ♀
Styria, Großlobming	N 47°11′, E 14°49′ (652 m)	10 May 2009	6 ♂, 6 ♀
Styria, near Großwilfersdorf, Feistritz (river) bank	N 47°04′51′′, E 15°59′09′′ (276 m)	27 November 2011	3 juv
		4 December 2012	2 juv
Styria, Hieflau	N 47°36′, E 14°43′ (510 m)	29 May 2010	1 ♀
Styria, near Lassing, Blosen	N 47°29′, E 14°16′ (1,025 m)	17 August 2008	2 juv
Styria, Wagna	N 46°45′, E 15°33′ (265 m)	28 April 2008	1 juv
Burgenland, near Eisenstadt, Gloriette Warte	N 47°51′19′′, E 16°30′11′′ (367 m)	20 April 2011	5 ♀, 1 juv

^a^Neither sex nor developmental stage determined

^b^Individuals from these collections were used to evaluate secretion profiles as described in the text

### Extraction of secretions

Scent gland secretions were obtained by three different methods: (1) directly by dabbing secretion on small pieces of filter paper (2 × 2 mm), immediately after emission from ozopores (subsequent to mechanical irritation of specimens by e.g., gentle squeezing). Secretion-loaded filter papers were extracted in hexane for 30 min; (2) by whole body extraction of individuals in 150 µl of hexane or methylene chloride for 30 min as already described and standardized for other harvestmen (e.g., Raspotnig et al. 2005, 2010); (3) by extraction of excised single glands in methylene chloride for 30 h.

It should be mentioned beforehand that the variation in secretion patterns as described in the results largely resulted from the generally problematic methodological access to the *Rilaena* secretion. In detail, the handling of specimens during extract preparation greatly affected the resulting profiles. Thus, for a reliable evaluation of the full secretion profile of *R. triangularis,* we exclusively used extracts prepared in a standardized manner as follows: Individuals were handed with extreme care to avoid early emission of secretion beginning with the very gentle handling of specimens in the course of collecting in the field. Individuals that could not be collected without disturbing/stressing them were excluded from the investigation. Also, to avoid effects of handling in the course of extract preparation in the laboratory, we excluded individuals that could not be transferred into the solvent rapidly, i.e. without stressing them for more than few seconds. Exposure to stress in any form (in particular mechanical stress when catching and holding the specimens) noticeably led to the premature emission of scent. When jet droplets were collected, we exclusively dabbed droplets immediately after emission from ozopores, disregarding droplets that already might have mixed up with enteric fluid.

### Analysis of extracts

Aliquots of extracts (1.5 µl) were subjected to gas chromatographic–mass spectrometric analysis. We used a Trace gas chromatograph coupled to a DSQI mass spectrometer (MS), both from Thermo (Vienna, Austria). The GC was equipped with a ZB-5MS fused-silica capillary column (30 m × 0.25 mm i.d., 0.25 µm film thickness, Phenomenex, Germany). Injection was splitless with helium (at 1.2 ml min^−1^) as a carrier gas. The column temperature was programmed from 50 °C (held for 1 min) to 300 °C at 10 °C min^−1^, and then held for 5 min. The ion source of the MS and the transfer line were kept at 200 and 310 °C, respectively. Electron impact (EI) spectra were recorded at 70 eV. For chromatographic reference, 1,4-benzoquinone, 1,4-naphthoquinone, caprylic (=octanoic) acid as well as a standard mixture of alkanes (C_9_–C_36_) were purchased from Sigma–Aldrich (Vienna, Austria). Derivatization of free n-octanoic acid from extracts to its trimethylsilyester was carried out by adding 50 µl of MSTFA (in Pyridin 2:1; with 1 % TMCS) to 50 µl of extract and by incubating the mixture at 60 °C for 30 min. Gas chromatographic retention indices (RI) of extract components were calculated using an alkane standard mixture, following the formula RI_*x*_ = 100*n*_0_ + (100t_*x*_− 100t*n*_0_)/(t*n*_1_–t*n*_0_), with *x* target compound; t_*x*_ retention time of target compound; *n*_0_ number of carbon atoms in the alkane directly eluting before *x*; t*n*_0_ retention time of alkane directly eluting before *x*; t*n*_1_ retention time of alkane directly eluting after *x*.

### Scanning electron microscopy

For scanning electron microscopy (SEM), specimens were fixed in Bouin, washed, dehydrated, air-dried and mounted onto aluminum stubs prior to sputtercoating with gold (AGAR sputtercoater, Gröpl, Tulln, Austria). Micrographs (SEM) were taken with a Philips XL30 ESEM (Philips/FEI, Vienna, Austria) at high vacuum mode and 20 kV accelerating voltage.

## Results

### Release of scent gland secretion

**Fig. 1:**
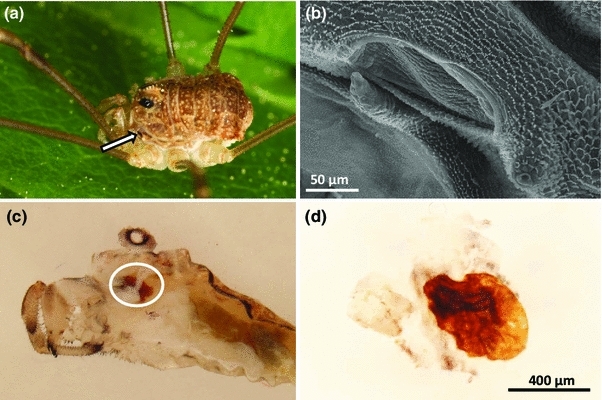
Scent glands of *Rilaena triangularis*. **a** Habitus of a female individual with ozopore (*arrow*) of left scent gland visible above coxa I. **b** Scanning electron micrograph of an ozopore. **c** Excision of scent glands: left gland appears as *brownish* sac-like structure (*encircled*) in this sagittal section. **d** Excised scent gland, showing *brownish* content

In case of mechanical irritation, *Rilaena triangularis* readily emits its scent gland secretion from ozopores near dorsal to coxae I (Fig. 1a, b). Secretion discharge was investigated in 36 individuals (12 males, 15 females, and nine juveniles). Following irritation (by moderately squeezing the body) 21 of these immediately emitted a colorless to slightly brownish secretion, mostly in the form of a jet (17 out of 21 individuals). The jet reached targets in a range of about 5 cm. The secretion itself exhibited a pungent smell. Emission of secretion, in particular jetting, could be evoked only once per individual. In most cases, the emission was paralleled by regurgitation of enteric fluid, and by spreading of a mixture of enteric fluid and scent gland secretion all over the body surface.

### Chemical identification of scent gland secretion compounds

**Fig. 2:**
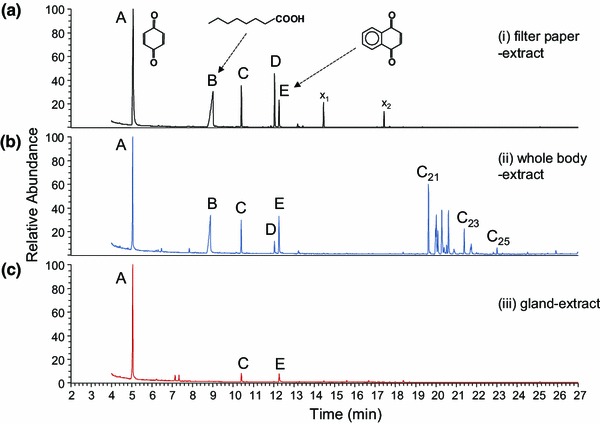
Chemistry of the *Rilaena* secretion. **a** Gas chromatographic profile of a jet droplet from a female specimen, extracted from a secretion-loaded filter paper piece, and assumingly representing the full scent gland secretion of *R. triangularis*. The profile exhibits five components: 1,4-benzoquinone (peak *A*), octanoic acid (peak *B*), unknown I (peak *C*), unknown II (peak *D*), and 1,4-naphthoquinone (peak *E*). Compounds *x*_1_ and *x*_2_ originate from the filter paper. **b** Profile of a whole body extract of a female specimen, showing all five peaks *A*–*E* from its putative scent gland secretion in a first chromatographic zone, and a number of cuticular hydrocarbons in a second chromatographic zone. Major hydrocarbons C_21_ (*n*-heneicosane), C_23_ (*n*-tricosane), and C_25_ (*n*-pentacosane) are indicated. **c** Profile of an extract of a single, excised scent gland, showing 1,4-benzoquinone (peak *A*), unknown I (peak *C*), and 1,4-naphthoquinone (peak *E*) only

Investigations into the chemistry of the *Rilaena*-secretion were performed with 147 individuals (39 males, 35 females, 31 juveniles; 42 adult individuals of undetermined sex; Table 1). Based on results from direct sampling of secretion, whole body extracts and extracts from excised glands, five compounds (peaks *A*–*E* in Fig. 2) were assigned to the scent gland products of *R. triangularis*. Details to extracts and to the different approaches toward secretion access are outlined in the chapter below.

The mass spectral fragmentation of compound *A* (M^+·^ at *m/z* 108) and compound E (M^+·^ at *m/z* 158) indicated 1,4-benzoquinone and 1,4-naphthoquinone (Table 2), both of which were readily identified by a comparison of gas chromatographic and mass spectrometric data to authentic standards. Compound *B* appeared to be a short chain aliphatic acid, exhibiting characteristic fragment ions at *m/z* 60 (base ion from McLafferty rearrangement) and at *m/z* 45 (from α-cleavage). From the expected series of [(CH_2_)_n_COOH]^+^-fragments, only those with *n* = 2 (at *m/z* 73) and *n* = 6 (at *m/z* 129) could be detected, the latter in very low relative intensity (about 1 %). A weak signal at *m/z* 144 appeared to be the molecular ion, and taken altogether, these data were consistent with a saturated C_8_-carboxylic acid. However, compound *B* did not elute in a sharp, but in a very broad peak, clearly depending on its concentration and hence resulting in poorly reproducible retention times. We nevertheless determined its retention index using a diluted sample (measured RI 1173). A literature survey for the RI of *n*-octanoic acid reflected this situation and a range of RIs from 1160 to 1190 has been reported for this compound (see NIST Chemistry Webbook at http://webbook.nist.gov/cgi/cbook.cgi?ID=C124072&Units=SI&Mask=2000#Gas-Chrom). For further clarification and to exclude the possibility of a branched isomer, we prepared the trimethylsilylester-derivative of the compound leading to the generation of a compound *B’* at higher retention time (measured RI 1267). Compound *B’* showed the expected increase in molecular weight by 72 amu (144 + C_3_H_8_Si), thus exhibiting a molecular ion at *m/z* 216 (C_11_H_24_O_2_Si^+·^) together with a characteristically intense M-15 fragment at *m/z* 201(100). Further fragment ions were consistent with an octanoic acid trimethylsilyester as well, and were observed at *m/z* 173(4), 157(7), 145(12), 132 (19), 131(19), 129(24), 117(65), 75(74), and 73(84). Both retention index and fragmentation pattern proved to be identical to data from *n*-octanoic acid-trimethylsilyester (e.g., Isidorov et al. 2005). Finally, synthetic *n*-octanoic acid and its trimethylsilylester revealed the same chromatographic and mass spectral characteristics as component *B* and *B’*, respectively, and co-injection of synthetic *n*-octanoic acid with one *Rilaena*-extract resulted in a single, but enlarged peak for compound *B*.

**Table 2:** Gas chromatographic and mass spectral data to components from scent glands of *Rilaena triangularis*

peak	retention index^a^	EI-fragmentation (*m/z*)		identified as
A	921	108 (M^+·^, 100), 82 (41), 80 (36), 54 (64), 52 (18)		1,4-benzoquinone
B	1173	144 (M^+·^, 1), 129 (1), 101 (20), 87 (13), 85 (19), 84 (20), 73 (70), 69 (12), 60 (100), 55 (30), 45 (11), 43 (43), 41 (36)		*n*-octanoic (=caprylic) acid
C	1280	184 (< 1), 141 (56), 123 (8), 81 (26), 71 (76), 67 (13), 57 (88), 55 (19), 43 (100), 41 (36)		unknown 1
D	1402	186 (< 1), 143 (14), 141 (30), 123 (4), 97 (6), 83 (52), 81 (13), 71 (29), 69 (100), 57 (60), 55 (59), 45 (23), 43 (57), 41 (36)		unknown 2
E	1419	158 (M^+·^, 100), 130 (34), 104 (37), 102 (45), 76 (45), 66 (8), 50 (25)		1,4-naphthoquinone

^a^RI, for calculation see “Materials and methods”

The two remaining extract-components *C* and *D* (both of which were minor components) could not yet be identified. Both components showed a similar mass spectrum, exhibiting (putative) molecular ions of low abundance at *m/z* 184 (compound *C*) and *m/z* 186 (compound *D*), respectively. Detailed analytical data to all five components *A*–*E* are given in Table 2.

### Scent gland origin of components

Extracts prepared by the three different methods (1–3, as specified in “Materials and methods”) indicated the following origins of our extract components (Fig. 2). (1) Direct support for the assignment of the five components *A*–*E* to the scent gland secretion of *Rilaena* came from individually dabbed secretion on filter-paper pieces, directly from ozopores (Fig. 2a). However, fluids at ozopores were sampled from 18 individuals, but only five extracts (=about 28 %) revealed the full pattern of all five components. The remaining extracts either showed major compounds *A*, *B*, and *E* only or did not exhibit any compounds at all. (2) For a more exhaustive access to the scent gland secretion of *R. triangularis* as well as for easier handling, we extracted scent gland secretions from whole bodies of freshly collected individuals (80 extracts, including 4 pools of 2–3 individuals). Whole body extracts led to chromatograms showing two distinct chromatographic zones (Fig. 2b). The first zone contained the already described array of putative scent gland components *A*–*E,* whereas a second chromatographic zone (zone II) showed up to 25 compounds. Compounds of zone II appeared to be common cuticular hydrocarbons all of which were present in both male and female extracts, though in different relative abundance (not shown). We identified the following zone II-components, mainly by comparison to a standard mixture of saturated *n*-alkanes: (1) five odd numbered *n*-alkanes, C_21_, C_23_, C_25_, C_27_, and C_29_. These represented the major zone II-compounds, with heneicosane (C_21_) being most abundant; (2) four even numbered *n-*alkanes C_22_, C_24_, C_26_, and C_28_. (3) Furthermore, we tentatively identified a number of branched alkanes on the basis of their mass spectra (data not shown), including three isomers with M^+·^ at *m/z* 310 (branched C_22_); four isomers with M^+·^ at *m/z* 324 (branched C_23_); four isomers with M^+·^ at *m/z* 338 (branched C_24_). With respect to the extract components of the first chromatographic zone, only 10 % of extracts showed the full set of zone I-components *A*–*E* (8 extracts out of 80). The majority of extracts (about 60 %) either exhibited a combination of 1,4-benzoquinone (compound *A*), octanoic acid (compound *B*), and 1,4-naphthoquinone (compound *E*) or even a combination of the two quinones only. In about 30 % of the extracts, no components of zone I but exclusively components of zone II could be detected (=no secretion components, empty glands). By contrast, zone II-compounds were consistently found in all whole body extracts. (3) To provide further direct evidence for the presence of compounds *A*–*E* in the scent gland reservoirs of *R. triangularis*, glandular reservoirs were dissected from frozen specimens (Fig. 1c). Scent gland reservoirs appeared to contain dark reddish content of granular consistence (Fig. 1d). Individual methylene chloride extracts of dissected single glands from 38 individuals consistently proved the presence of 1,4-benzoquinone as major extractable component (Fig. 2c). In addition, in two extracts of excised glands, small amounts of 1,4-naphthoquinone were detected as well; in one case together with component *C*. None of the glandular extracts contained octanoic acid or compound *D*.

### Secretion patterns

Secretion profiles were evaluated from 12 “good” extracts (four filter-paper extracts from direct sampling and eight whole body extracts, all from adult individuals) all of which showed the full set of the five scent gland components *A*–*E* in large amounts. Profiles were calculated based on the relative abundance of single compounds (% peak area of total secretion), proving 1,4 benzoquinone as the main component of the secretion (amounting for 46.4 ± 16.1 %), followed by octanoic acid (32.5 ± 11.9 %) and 1,4-naphthoquinone (7.4 ± 3.4 %). Components *C* and *D* were less abundant (compound *C*: 6.9 ± 2.8 %; compound *D*: 6.9 ± 3.3 %). We did not find differences between male and female secretions. A preliminary approach on juveniles (no secretion profiles shown) indicated the presence of the three major compounds *A*, *B*, and *E* whereas compounds *C* and *D* were not detected in any extract.

## Discussion

### The elusive scent gland secretion of Rilaena triangularis

Our attempts toward collection/extraction of the *Rilaena*-secretion turned out to be much more difficult than in any other harvestmen species previously investigated, and we indeed needed 100 individuals to “learn” the extraction procedure in a fairly reproducible manner. Secretion emission occurs rapidly, frequently already in the course of collection of individuals, and such stressed individuals were found to contain residual secretion only or no secretion at all. Also, jets produced by stressed individuals frequently mixed up with enteric fluid, wetting the individual completely, and thus making it difficult to dab pure secretion. However, we consider all five components of chromatographic zone I in our extracts, i.e. 1,4-benzoquinone, octanoic acid, 1,4-naphthoquinone and the two unknown compounds *C* and *D* to represent constituents of the scent gland secretion of *R. triangularis*. A strong argument for this is their occurrence in ejected liquid directly dabbed from ozopores. Moreover, 1,4-benzoquinone was found by all extraction methods applied, and could consistently be detected in extracts of excised glands as well. The brownish color of the glands may be due to the mix of 1,4-benzoquinone (yellow) and 1,4-naphthoquinone (reddish). The latter compound could be detected in the extracts of excised glands as well, though sporadically only. We consider this phenomenon rather a methodological problem of naphthoquinone-solubility (is poorly soluble) and its putative location within the gland. Though glandular reservoirs are quickly emptied, preliminary data indicate that large amounts of brownish material still remain between the top of the epithelial cells of the reservoir and the covering intima. The intima certainly represents a barrier hindering the passage of secretion, particularly with respect to poorly soluble constituents. We also consider the two unknowns *C* and *D* to be a part of the scent gland secretion: at least compound *C* was found in extracts of excised glands. We think that both compounds may be involved in the solubilization process of the quinones and that they may be located in the cavity of the reservoirs from which frequently only remains could be extracted. Octanoic acid, on the other hand, was found in both whole body extracts and in jets, but could not be extracted from excised glands. Like the unknown compounds *C* and *D*, octanoic acid may be considered a part of the filling of the glandular reservoirs, and thus it may be not easily extractable from excised (and already emptied) glands.

### Palpatorean benzoquinones and their phylogenetic significance

With respect to scent gland-based chemosystematics (e.g., Raspotnig 2012, 2014b), the most surprising finding of the present study is certainly the presence of 1,4-benzoquinone in the scent glands of a representative of Eupnoi. We know that benzoquinones are characteristic exocrine compounds of many arthropods, and that these are particularly widespread in the defensive glands of diplopods, beetles, dermapterans, and laniatorean harvestmen (e.g., Blum 1981; Eisner et al. 2000; Deml and Huth 2000; Dettner 1987, 1993; Bodner and Raspotnig 2012; Rocha et al. 2013). In terms of “semiochemical parsimony” (sensu Blum 1996), the allomonal properties of benzoquinones, in particular their potency of deterring predators (e.g., Eisner et al. 2004; Machado et al. 2005), probably made these substances well suited for chemical defense, favoring their multiple convergent evolution in a variety of taxa. However, the defensive secretions of some particular taxa exclusively rely on benzoquinones, such as the secretions from serial defensive glands of juliform diplopods (“quinone millipedes” sensu Eisner et al. 1978) or the benzoquinone-rich scent gland secretions of already mentioned higher gonyleptoid harvestmen (e.g., Föttinger et al. 2010; Caetano and Machado 2013). Within such taxa, a singular evolutionary origin of benzoquinones is indicated, and appears to be a likely and parsimonious explanation for benzoquinone distribution.

We here finally provide evidence that scent gland-derived benzoquinones indeed occur in palpatorean Eupnoi as well, with *R. triangularis* representing only a first example. According to our preliminary data from other Eupnoi, the production of 1,4-benzoquinone is indeed more widespread and seems to be a distinguishing character of certain phalangiids, particularly characterizing representatives of subfamily Platybuninae (at least genera *Megabunus*, *Platybunus*). Upon the example of *Rilaena*, we eventually show that benzoquinones evolved at least twice independently in the scent glands of Opiliones, once in Laniatores, and once in phalangiid Eupnoi. Neither there is evidence for a close phylogenetic relationship of phalangiids to Laniatores, nor for a consistent benzoquinone-producing lineage from palpatoreans to laniatoreans. Thus, a common ancestry of harvestman benzoquinone-production can de facto be excluded.

In addition, there are major structural differences between palpatorean and laniatorean benzoquinones, possibly indicating different biosynthetic pathways, and thus supporting a double evolutionary origin as well. From laniatoreans, exclusively alkylated benzoquinones have been reported, whereas in *Rilaena* (and also in other Platybuninae: Raspotnig, unpublished) the exudate is composed of non-substituted 1,4-benzoquinone. As recently demonstrated by Rocha et al. (2013), benzoquinone biosynthesis in Laniatores relies on the formation of a polyketid chain from acetate/mevalonate, followed by cyclization, resulting in alkylated phenols that subsequently are oxidized to alkylated benzoquinones. Laniatorean benzoquinones are thus clearly derived from the ancestral state of phenol-rich secretions (as present in lower grassatoreans such as Phalangodidae), and most likely have been invented first in a clade of ancient gonyleptoids after the split-off of the basal gonyleptoid (and still phenol-producing) family Stygnopsidae (Sharma and Giribet 2011; Rocha et al. 2013; Raspotnig et al. 2014b). In *Rilaena* (and other Eupnoi) no phenolic precursors have been detected, and it is currently not clear how eupnoan 1,4-benzoquinone is synthesized. It is well known, however, that different pathways could lead to benzoquinones in arthropods. According to Morgan (2004), one of these pathways relies on acetate condensation (the polyketid pathway as in laniatoreans), another one requires tyrosine and may lead to benzoquinone itself (e.g., Meinwald et al. 1966).

### Scent gland secretion of Rilaena and its placement among phalangiid secretion chemistry

Adding to a single study on *Phalangium opilio* (Wiemer et al. 1978), we here present a second example for the scent gland chemistry of the Phalangiidae. There is an early stated rough chemical dichotomy among eupnoan secretions, on the one hand acycles in Sclerosomatidae, and on the other hand naphthoquinones in Phalangiidae (Wiemer et al. 1978; Ekpa et al. 1985). At least superficially, our data may be considered support for this hypothesis. The secretions of both, *P. opilio* and the herein studied *R. triangularis* may be classified as of the “naphthoquinone” type. From preliminary studies, we know that by far not all phalangiid secretions rely on naphthoquinones. On the level of Palpatores, naphthoquinone-rich secretions are known from nemastomatid Dyspnoi (Raspotnig et al. 2010, 2014a) appear to be scattered across phalangiid Eupnoi (Wiemer et al. 1978; Raspotnig 2012) but may generally lack in Sclerosomatidae (Ekpa et al. 1985). A chemosystematic evaluation of this situation must consider the bias between the de novo-evolution of a compound (requiring a biosynthetic multistep-machinery, and thus many steps) and the reduction of a compound (may happen by the loss/inactivation of a single enzyme, thus possibly requiring only one step) (Raspotnig et al. 2014b). In these terms, eupnoan naphthoquinone distribution is more likely explained by multiple independent events of reduction of a plesiomorphic palpatorean naphthoquinone matrix than by the much less parsimonious multiple independent evolution of naphthoquinone production.

Regarding internal phalangiid systematics, we consider scent gland-derived chemical data as a very useful tool for an already overdue systematic revision of the family Phalangiidae. Phalangiid systematics is highly artificial (e.g., Tsurusaki 2007) and would clearly benefit from the inclusion of an independent data set to data from conventional sources. Four subfamilies (Phalangiinae, Platybuninae, Opilioninae, Oligolophinae) and the so-called “*Dicranopalpus* group” are currently recognized, but relationships among these subfamilies and the delimitations of subfamilies are still uncertain or tentative. Data on *Rilaena* and preliminary data on other phalangiids indicate high chemical diversity along with highest specificity of phalangiid secretions. For instance, in *P. opilio,* the secretion is described to consist exclusively of 1,4-naphthoquinone and 6-methyl-1,4-naphthoquinone (Wiemer et al. 1978), clearly distinct from the chemically more diverse secretion of *Rilaena*.

Particularly the co-occurrence of benzoquinone and naphthoquinone in *Rilaena* is a highly unusual feature, rarely realized in arthropods (e.g., Tschinkel 1972; Deml and Huth 2000), and not a single case for benzo- and naphthoquinone co-occurrence has so far been reported from the Opiliones. In this respect, *R. triangularis* is a key species of the family Phalangiidae: *Rilaena triangularis* has been transferred to subfamily Phalangiinae (Starega 1973) after its initial placement in Platybuninae (Dumitrescu 1970). However, the species resembles platybunines in several aspects. Also chemically, *R. triangularis* (together with a yet undefined part of Platybuninae) belongs to a possibly monophyletic benzoquinone-producing lineage among the Phalangiidae, linking the naphthoquinone-producing Phalangiinae to benzoquinone-emitting Platybuninae.
